# Gender-dependence of substituted judgment on quality of life in patients with dementia

**DOI:** 10.1186/1471-2377-11-118

**Published:** 2011-09-30

**Authors:** Claudia Schiffczyk, Christina Jonas, Constanze Lahmeyer, Friedemann Müller, Matthias W Riepe

**Affiliations:** 1 Department of Psychiatry and Psychotherapy II, Mental Health & Old Age Psychiatry, Ulm University, Ulm, Germany; 2 Alzheimer Therapiezentrum Bad Aibling, Bad Aibling, Germany

## Abstract

**Background:**

Substituted judgment asks the proxy to decide what the patient would have decided, had he or she been competent. It is unclear whether substituted judgment of the patient's quality of life can serve as a surrogate measure in patients with dementia.

**Methods:**

212 patients with dementia and their proxies were interviewed in their homes. Dementia syndrome was characterized with cognitive, non-cognitive and functional scales. Quality of life (QoL) was assessed with the QoL-AD.

**Results:**

Substituted judgment of the patient's QoL was unrelated to dementia severity but also correlated with the proxie's own QoL (r = 0.356; p < 0.001). Gender-specific analysis reveals that for male proxies the most important variable is severity of patient's depression (r = -0.895; p = 0.001) while for female proxies it is the proxie's own QoL (r = 0.371; p < 0.001). Subjective burden correlates with the proxie's QoL in females (r = -0.282; p = 0.001) but not in males (r = -0.163, p = 0.161).

**Conclusion:**

Substituted judgment of the patient's QoL does not correlate with dementia severity. Substituted judgment is subject to proxy-related variables in a gender-dependent fashion and therefore not suited to serve as an appropriate surrogate of the patients' quality of life.

## Background

Alzheimer's disease (AD) is a neurodegenerative disease with increasing prevalence in the aging societies of the Western hemisphere. Similarly, the prevalence of other age-related diseases increases. For all these diseases and conditions therapeutic interventions are being developed and improved. Common outcome variables are requested by today's health care systems to allow a comparison of the impact of diseases and the efficacy of treatments. One widely considered option to analyze the impact of disease on patients' life is to evaluate "Quality of life" (QoL).

The World Health Organisation defines QoL as "the individual's perceptions of their position in life in the context of the culture and value systems in which they live, and in relationship to their goals, expectations, and standards" (WHO-QoL, 1995). By this definition QoL is a subjective construct, being evaluated by the affected person by means of self reports. A widely used model assumes four domains which contribute to the individual's QoL: behavioural competence, psychological well-being, objective environment, and self-perceived QoL [1]. Hence, it is widely accepted that QoL is a multidimensional construct containing both subjective and objective elements, such as perceived contentment and functional abilities, respectively. Intuitively, one would assume, that dementia severity is inversely correlated with the self assessed Quality of life of the patient. However, the burden imposed by disease may be perceived differently in varying stages of disease which could result in a bi- or multimodal distribution of perceived quality of life over different stages of disease severity. Indeed, the validity of self-assessment of quality of life in dementia patients has recently been questioned [2-4].

Compared with their proxies, patients in early stages of dementia are likely to give overly optimistic ratings of their own mental capacities, lost functions, activities and social relationships [5]. Therefore clinicians and clinical investigators often rely on external evaluation of the patient by the caregiver and use this information as a substitute.

Substituted decision making for patients who lack capacity, however, is a much-discussed topic. Substituted judgment implies that the substitute for the incompetent patient is the decision given by proxy considering what the patient would have decided, had he or she been competent [6]. However, agreement between the patient's and the substitute's decision in several studies has shown to be mediocre at best, and it has been debated whether substituted judgment is an adequate surrogate for the patient's decisions [7,8]. Discussion between patient and proxy has been found to facilitate more accurate substituted judgment regarding the preferences of patients for life-sustaining therapies [8]. Overall, the accuracy of substituted judgments is subject to the kind of scenario to be decided, the amount of discussion between patient and surrogate and multiple clinically apparent patient- and proxy-related factors [9].

Dementia results in impaired cognition, language, insight, and judgment, and other behavioural and psychological symptoms of dementia (BPSD). Therefore, proxies often make decisions on the patient's behalf. Proxies are even used as informants in clinical studies on therapeutic efficacy. This assumes that proxies describe the patient's behaviour and well-being in an objective and reliable fashion. Theoretically, the proxy should be able to know the preferences of the patient, considering that dementia often extends over a long time giving proxy and patient plenty of time to discuss preferences. Empirically, however, proxy-ratings of QoL in AD patients does not correlate well with the patients' own answers, challenging the validity of the reported answers by the caregivers [10-12].

In the present study in patients' and proxies' homes, the proxy was asked to rate the QoL of the patient as a substituted judgment, i.e. how the patient would assess his or her own QoL should he or she be competent. It was the goal to analyze whether substituted judgment of the patients' quality of life can serve as a surrogate measure of the patients' quality of life or whether it is modulated by proxy-related variables.

## Methods

The study was performed according to institutional guidelines and the principles laid out in the Declaration of Helsinki. All patients and proxies gave their written consent to participate in this study.

### Patients and Caregivers

The patients and their proxies were recruited from a cohort of patients interested in or applying for a short-term in-patient treatment at the Alzheimer Therapy centre Bad Aibling. Most of the dyads applying were spouse dyads (see below) which limits the generalizability of the study. There may be other recruitment biases involved but the nature of the study did not allow the systematic control of other possible biases. It can be ruled out, however, that these patients represent a selection of persons with acute behavioural and psychological symptoms of dementia, as the time between application for short-term rehabilitation and approval by the health insurance takes several weeks on average. Initial contact and screening of eligibility to take part in the study were made via telephone by a study nurse. Criteria for inclusion in the study were a diagnosis of dementia of a mixed or Alzheimers type performed by a general practitioner or neurologist/psychiatrist. Only patients living in one household with their primary proxy were included in the study. The primary proxy was the spouse in 180 of 194 dyads (93%). This predominance of spouse dyads limits the generalizability of the study to dyads with other patient-proxy relations.

**Figure 1:**
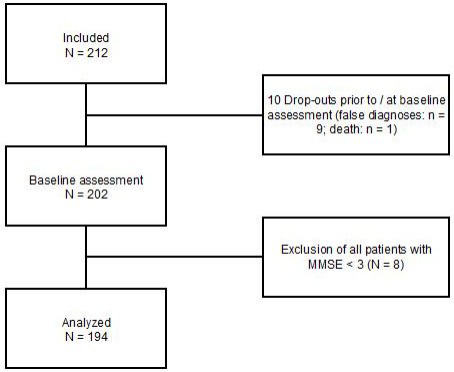
**Flow chart of the study cohort**.

Baseline assessment was performed between September 2008 and June 2010 with a MMSE score of 3 and above. Figure 1 shows the flow chart of the study cohort.

The remaining sample comprises 194 patients with either AD or mixed dementia (mean age 73.0 ± 7.1 years, range 52 - 89 years; 70,6% male) and their proxies (mean age 69.0 ± 7.7 years, range 43 - 90 years; 27.8% male). MMSE scores ranged from 3 - 28 (mean 17.3 ± 6.8).

All interviews took place in the domestic surroundings of these families after explaining the aim of the study and obtaining informed consent by both the patient and the proxy. Assessments were carried out by specially trained research assistants. Patients and proxies were interviewed separately to minimize bias and mutual influence on the responses.

### Nomenclature

In order to make clear who the person was that performed the scale/questionnaire and whom that person rated we used the following nomenclature: rater(measure)rated person. For example: proxy(QoL-AD substituted)patient means that the proxy rated the patient's QoL as the patient would have done, and proxy(QoL-AD)proxy signifies that the proxy rated his or her own QoL.

### Assessments

#### Mini-Mental Status Examination (MMSE [13])

The MMSE is the most commonly used instrument to stage the severity of dementia by assessing cognitive functions. It comprises tests on orientation, registration, short-term memory, language use, comprehension, and basic motor skills. Scores range from 0 - 30. Commonly, the scores on the Mini-Mental Status Examination are used to describe the severity of dementia. Patients are considered to be in mild stages of disease when scoring 20 points or above, to be in moderate stages of disease when scoring between 10 and 19, and severe when scoring 9 or less. The test was administered to patients, only.

#### Behavioural pathology in Alzheimer's Disease Rating Scale (Behave-AD [14])

The Behave-AD is a clinical rating instrument to characterize the phenomenology of behavioural symptoms. It comprises 25 items, all of which are answered by the proxy. The test was administered to proxies, only, to characterize behavioural symptoms of the patients (proxy(Behave-AD)patient).

#### Geriatric Depression Scale (GDS [15])

The Geriatric Depression scale uses a 15-item questionnaire to assess symptoms of depression and has been validated in the cognitively intact and the demented elderly [16,17]. The test was administered to both, patients and proxies (proxy(GDS)proxy and patient(GDS)patient, respectively).

#### Activities of Daily living (B-ADL [18])

This scale is used to assess the deficits in the performance of the patients' everyday activities. It comprises 25 items, all of which are answered by the proxy. Ratings are made on a 10-point Likert-type scale. The test was administered to proxies, only, to characterize activities of daily living of the patients (proxy(B-ADL)patient).

#### Instrumental Activities of Daily living (iADL [19])

This scale is used to assess the deficits in the performance of the patients' instrumental activities of daily living. It comprises 8 items with lower scores indicating worse performance in activities. The test was administered to proxies, only, to characterize instrumental activities of daily living in the patients (proxy(iADL)patient).

#### Euro-QoL (EQ-5D [20])

The EQ-5D questionnaire is a generic instrument to measure health related QoL in five domains: mobility, self-care, usual activities, pain/discomfort and anxiety/depression. It can be applied to patients as well as used in proxies to rate their own and the patients' QoL [21,22]. There are two core components of this instrument: a description of the respondents own health in the above mentioned five domains (rated on a three point Likert scale each) and a rating of their overall own health on a visual analogue scale (VAS, score 0 - 100). In order to capture the cognitive deterioration, a cognitive dimension was added (EQ-5D+C). The test was administered to both, proxies and patients. In this manuscript we only analyzed the EQ-5D VAS of the proxy (proxy(EQ-5D VAS)proxy).

#### Quality of Life in Alzheimer's disease (QoL-AD)[23]

The QOL-AD is a 13-item questionnaire on Quality of life and can be used both in patients with dementia [24] and healthy elderly controls [25]. The questionnaire covers the following domains: physical health, energy, mood, living situation, memory, family, marriage, friends, chores, fun, money, self and life as a whole. The QoL-AD is structured in a four-choice format, ranging from 1 being poor and 4 being excellent and refers to the patient's *current *QoL. Possible scores range from 13-52 with higher scores indicating better QoL. In this study we administered the scale to the caregiver to measure their own QoL (proxy(QoL-AD)proxy), as well as the patients' QoL via substituted judgment (proxy(QoL-AD substituted)patient). A substituted judgment asks the caregiver to rate how they think the patient views his or her own QoL. Subsequent to reading each item the proxy was reminded to provide the answer that he or she thinks the patient would give.

#### Frequency of medication use

The proxy was asked about the daily intake of medication by himself or herself and about the frequency of the patient with a fourfold choice (1: no medication use; 2: 1 to 3 times; 3: 4 to 6 times; 4: more than 6 times). Funding of the study did not allow to assess symptoms of neuropsychiatric disease or multimorbidity in the proxies in detail. However, when designing the study we aimed at having at least one indirect measure of multimorbidity in patients and proxies and used frequency of medication use as a surrogate of multimorbidity.

### Data Analysis

All statistical analyses for the investigation of group differences were carried out using the statistics program SPSS (SPSS 15.0 for Windows, Chicago, Ill., 2001).

## Results

### Quality of life - the caregiver's self-assessment (proxy(QoL-AD)proxy)

Not surprisingly, less behavioural symptoms of the patients are associated with higher scores in the proxy(QoL-AD)proxy scale (proxy(Behave-AD)patient: r = -0.278; p < 0.001). Moreover, the score of the proxy(QoL-AD)proxy is correlated with his or her self-assessed mood (proxy(GDS)proxy: r = -0.635; p < 0.001) and his or her self-reported overall health (proxy(EQ-5D VAS)proxy: r = 0.459; p < 0.001).

To get further insight into the caregiver's variables we performed a gender-specific analysis. Severity of the patient's dementia was alike for male and female caregivers (MMSE 17.37 ± 6.56 and 17.10 ± 6.89, respectively, p = 0.800). Gender-specific analysis reveals that mood and overall health correlate with the proxy(QoL-AD)proxy score in both male and female caregivers (proxy(GDS)proxy: males: r = -0.528, p < 0.001; females: r = -0.646, p < 0.001 and proxy(EQ-5D VAS)proxy: males: r = 0.288, p = 0.035; females: r = 0.471, p < 0.001). Despite the overall association of mood with the proxy(QoL-AD)proxy score in both males and females, depression itself is gender-dependent (proxy(GDS)proxy: males: 2.66 ± 2.60, females: 3.91 ± 2.84, p = 0.005). Subjective QoL of the caregiver (proxy(QoL-AD)proxy) is associated with functional assessment of the patient by the caregiver, although only in female caregivers (females: r = -0.241, p = 0.004; males: r = -0.157, p = 0.258). The burden experienced by the proxy was slightly higher in females than in males (2.14 ± 0.87 vs. 1.81 ± 1.03, respectively, p = 0.046). The burden correlates with the proxy(QoL-AD)proxy score in females (r = -0.282; p = 0.001) but not in males (r = -0.163, p = 0.161). Data are displayed in Table 1.

**Table 1:** Gender-specific analysis of patient and proxy-related variables grouped according to sex of the proxy.

		male proxies	female proxies
		n = 54	n = 140
*Proxy-related variables*			
Age	mean	72.43	67.63
	SD	6.64	7.67
	t-test	p < 0.001	
proxy(GDS)proxy	mean	2.66	3.91
	SD	2.60	2.84
	t-test	p = 0.005	
proxy(QoL-AD)proxy	mean	38.35	35.95
	SD	4.31	5.36
	t-test	p = 0.002	
***Patient's self report***			

Age patient	mean	71.70	73.52
	SD	6.66	7.20
	t-test	p = 0.099	
MMSE	mean	17.37	17.10
	SD	6.56	6.89
	t-test	p = 0.800	
patient(GDS)patient	mean	3.09	2.84
	SD	3.19	2.60
	t-test	p = 0.605	
***Proxie's report about patient***			

proxy(Behave-AD)patient	Mean	5.22	6.44
	SD	4.88	4.76
	t-test	p = 0.121	
proxy(B-ADL)patient	Mean	7.00	7.68
	SD	2.67	2.04
	t-test	p = 0.100	
proxy(iADL)patient	Mean	3.39	2.50
	SD	2.52	1.87
	t-test	p = 0.021	

### Quality of life - substituted judgment (proxy(QoL-AD substituted)patient)

The proxy was asked to rate the QoL of the patient as a substituted judgment (proxy(QoL-AD substituted)patient), i.e. how the patient would assess his or her own QoL would he or she be competent. Similar results as in the self-assessment were obtained for the scores of the substituted judgment (proxy(QoL-AD substituted)patient): (proxy(Behave-AD)patient: r = -0.308; p < 0.001); (proxy(GDS)proxy: r = -0.299; p < 0.001); (proxy(EQ-5D VAS)proxy: r = -0.203; p < 0.001). Spearman's rho for the correlation between proxy(QoL-AD)proxy and proxy(QoL-AD substituted)patient ranged from -0,005 and 0,490 for the individual items of the QoL-AD scale, indicating that the proxy judged differently when asked about himself or herself and asked to serve as a substitute of the patient (Table 2).

**Table 2:** Correlation between proxy(QoL-AD)proxy and proxy(QoL-AD substituted)patient for the individual items.

Individual Items Qol-AD	Spearman's rho	p
Physical Health	0.137	0.085
Energy	0.185	0.010*
Mood	0.146	0.042*
Living situation	0.159	0.027*
Memory	-0.005	0.948
Family	0.477	< 0.001*
Marriage	0.490	< 0.001*
Friends	0.477	< 0.001*
Self	0.095	0.187
Household chores	0.074	0.305
Do things for fun	0.219	0.002*
Money	0.405	< 0.001*
Life as a whole	0.250	< 0.001*

Commonly, severity of dementia is staged according to the score in the MMSE (mild: MMSE score ≥ 20; moderate: 10 ≤ MMSE score ≤ 20; severe: 3 ≤ MMSE score ≤ 9). The proxy(QoL-AD substituted)patient score was unrelated to severity of dementia (Table 3).

**Table 3:** Patient and proxy-related variables grouped according to severity of dementia.

		mild	moderate	severe
*Patient-related variables*				
	N	81	83	30
Age patient	Mean	72.91	73.24	72.67
	SD	7.06	7.07	7.41
	one-way ANOVA		p = 0.918	
MMSE	Mean	23.67	14.87	6.03
	SD	2.48	2.83	2.25
	one-way ANOVA		p < 0.001	
***Patient's self report***				

proxy(GDS)proxy	Mean	2.93	2.99	2.63
	SD	2.94	2.77	2.31
	one-way ANOVA		p = 0.844	
***Proxy-related variables***				

Age proxy	Mean	68.86	69.40	68.03
	SD	8.00	7.58	7.26
	one-way ANOVA		p = 0.701	
proxy(QoL-AD)proxy	Mean	36.68	36.72	36.17
	SD	4.99	5.93	3.35
	one-way ANOVA		p = 0.874	
proxy(GDS)proxy	Mean	3.35	3.70	3.80
	SD	2.77	2.85	2.95
	one-way ANOVA		p = 0.649	
***Proxie's report about patient***				

proxy(Behave-AD)patient	Mean	4.68	7.04	7.33
	SD	4.46	4.59	5.47
	one-way ANOVA		p = 0.002	
proxy(B-ADL)patient	Mean	6.36	8.19	8.57
	SD	2.49	1.69	1.49
	one-way ANOVA		p < 0.001	
proxy(iADL)patient	Mean	3.93	2.00	1.63
	SD	2.38	1.27	1.59
	one-way ANOVA		p < 0.001	
***Proxie's substituted report***				

proxy(QoL-AD substituted)patient	Mean	33.58	33.25	32.67
	SD	5.07	6.39	5.28
	one-way ANOVA		p = 0.752	

*Mini-Mental Status Examination *[13] (MMSE; mild: MMSE ≥ 20; moderate: 10 ≥ MMSE ≤ 19; severe:

MMSE ≤ 9) of the patient. GDS: *Geriatric Depression Scale*; QoL-AD: *Quality of Life in Alzheimer's*

*disease*; Behave-AD: *Behavioral pathology in Alzheimer's Disease Rating Scale *[14]; B-ADL:*Activities*

*of Daily living scale *[18]; iADL: *Instrumental Activities of Daily living *[19]

To analyze which variables related to the severity of the disease of the patient, or which caregiver-related variables relate to the substituted judgment on the quality of life of the patient by the proxy (proxy(QoL-AD substituted)patient), we performed a stepwise regression analysis. As independent variables characterizing the severity of the disease of the patient we used the total scores of the MMSE, the patient(GDS)patient, the proxy(Behave-AD)patient, the proxy(B-ADL)patient, the proxy(iADL)patient, and the frequency of drug use of the patient. As variables characterizing the well-being and health of the proxy, we used the total score of the proxy(EQ-5D VAS)proxy and the proxy(QoL-AD)proxy. Both age of the patient and age of the proxy were included as independent variables in the analysis. The most important variables contributing to the caregivers' substituted judgment of the patients' QoL (proxy(QoL-AD substituted)patient) were depression of the patient, self-rated QoL of the caregiver (proxy(QOL-AD)proxy), and neuropsychiatric symptoms of the patient. In the whole group as well as in the group with male or female proxies, these three variables explain about one third of the variance of substituted QoL-appraisal by the proxy. Data are displayed in Table 4.

**Table 4:** Stepwise regression of substituted judgment of the patients' QoL (proxy(QoL-AD substituted)patient).

R^2^	degrees of freedom	change in F	overall significance		Regression coefficient	T	significance
***All proxies***							

0.127	1,192	27.946	p < 0.001	**proxy(QoL-AD)proxy**	0.390	5.286	p < 0.001
0.213	1,191	20.822	p < 0.001	**proxy(QoL-AD)proxy**	0.373	5.299	p < 0.001
				**patient(GDS)patient**	-0.608	-4.563	p < 0.001
0.290	1,190	20.632	p < 0.001	**proxy(QoL-AD)proxy**	0.313	4.581	p < 0.001
				**patient(GDS)patient**	-0.582	-4.583	p < 0.001
				**proxy(Behave-AD)patient**	-0.335	-4.542	p < 0.001
***Male Proxies***							

0.200	1,52	12.962	p = 0.001	patient(GDS)patient	-0.895	-3.600	p = 0.001
0.377	1,51	14.515	p < 0.001	**patient(GDS)patient**	-0.940	-4.241	p < 0.001
				**proxy(QoL-AD)proxy**	0.618	3.810	p < 0.001
0.441	1,50	5.710	p = 0.021	**patient(GDS)patient**	-0.864	-4.026	p < 0.001
				**proxy(QoL-AD)proxy**	0.559	3.556	p = 0.001
				proxy(Behave-AD)patient	-0.335	-2.390	p = 0.021
***Female proxies***							

0.134	1,138	21.382	p < 0.001	proxy(QoL-AD)proxy	0.371	4.624	p < 0.001
0.218	1,137	14.736	p < 0.001	**proxy(QoL-AD)proxy**	0.315	4.043	p < 0.001
				**proxy(Behave-AD)patient**	-0.337	-3.839	p < 0.001
0.258	1,136	7.286	p = 0.008	**proxy(QoL-AD)proxy**	0.293	3.821	p < 0.001
				**proxy(Behave-AD)patient**	-0.337	-3.921	p < 0.001
				**patient(GDS)patient**	-0.423	-2.699	p = 0.008
0.293	1,135	6.622	p = 0.011	**proxy(QoL-AD)proxy**	0.290	3.864	p < 0.001
				**proxy(Behave-AD)patient**	-0.335	-3.988	p < 0.001
				**patient(GDS)patient**	-0.399	-2.593	p = 0.011
				**Age proxy**	0.132	2.573	p = 0.011

### Factor analysis of QoL-AD substituted judgment (proxy(QoL-AD substituted)patient)

To compare the dimensions of importance in answering the QoL-AD questionnaire when proxies report for themselves (proxy(QoL-AD)proxy), and when they perform substituted judgment (proxy(QoL-AD substituted)patient) a factor analysis was performed with a principal factor analysis of the QoL-AD item-level data, using an Oblimin-direct rotation with eigenvalues greater than 1 to sufficiently consider partial correlation between factors [26]. All analyses (total group, male proxies, and female proxies) had a Kaiser-Meyer-Olkin (KMO) measure between 0.8 and 0.9 exceeding the size of 0.5 or greater recommended for meaningful factor analysis [26]. For interpretation of the rotated factors, salient loadings were defined as values greater or equal to 0.5; in cases where an item did not have a loading of 0.5 or greater, its highest loading was selected to define its position in the factor structure.

Overall, the factor structure for self-assessment and for substituted judgment resembles these mutually within the whole group as well as the gender specific subgroups. Item "money" is somewhat important in the proxies' self assessment (proxy(QoL-AD)proxy)but is of no sigificance in the substituted judgment (proxy(QoL-AD substituted)patient). Similarly, item "memory" is of some importance in the proxies' self assessment (proxy(QoL-AD)proxy but less so in the substituted judgment(proxy(QoL-AD substituted)patient). General health is of similar importance in the whole group and for both males and females. In the male proxies' substituted judgment (proxy(QoL-AD substituted)patient) this comprises "household chores" and "doing things for fun". These items make up a separate factor in the female proxies' substituted judgment (proxy(QoL-AD substituted)patient). Moreover, "marriage" is a separate factor in the male proxies' substituted judgment (proxy(QoL-AD substituted)patient), whilst in the female proxies' judgment (proxy(QoL-AD substituted)patient) it is combined with item "family" and "life as a whole". Results of the factor analysis of the proxies self-judgment on their QoL are shown in Table 5, results for proxies' substituted judgment of patients' QoL are shown in Table 6.

**Table 5:** Factor-analysis for self-judgment of QoL (proxy(QoL-AD)proxy) in proxies

	All proxies - proxy(QoL-AD)proxy			Male proxies - proxy(QoL-AD)proxy					Female proxies - proxy(QoL-AD)proxy		
	**Factor**			**Factor**					**Factor**		

	**1**	**2**	**3**	**1**	**2**	**3**	**4**	**5**	**1**	**2**	**3**

**Physical Health**	,519			,776							,499
**Energy**	,566			,589					,494		
**Mood**	,727			,605					,735		
**Living situation**	,718			,508					,710		
**Memory**			,429			,853					,460
**Family**		,457	,418							,688	
**Marriage**								,492		,404	
**Friends**		,436			,585						
**Self**	,815			,663					,713		
**Household chores**			,435					-,586			,466
**Do things for fun**	,424				,770				,416		
**Money**			,480				,708				
**Life as a whole**	,721				,811				,834		

**Table 6:** Factor-analysis for proxies' substituted judgment of patients' QoL (proxy(QoL-AD substituted)patient)

	All proxies - proxy(QoL-AD substituted)patient			Male proxies - proxy(QoL-AD substituted)patient			Female proxies - proxy(QoL-AD substituted)patient		
	**Factor**			**Factor**			**Factor**		

	**1**	**2**	**3**	**1**	**2**	**3**	**1**	**2**	**3**

**Physical Health**	,683			,739			,651		
**Energy**	,535			,840			,549		
**Mood**	,710			,785			,673		
**Living situation**	,697			,758			,679		
**Memory**					,461		,462		
**Family**		,488						,563	
**Marriage**		,505				,817		,542	
**Friends**					,863				
**Self**	,502			,628			,496		
**Household chores**			-,693	,728					,684
**Do things for fun**			-,660	,533					,623
**Money**	,683								
**Life as a whole**	,535			,697			,510	,455	

## Discussion

### Patients and proxies

Similar to other reports in the literature [27], the male proxies of dementia patients who sought help were older than their female counterparts applying for short-term rehabilitation. However the age of the patient cared for was not gender-specifically different.

Depression is a frequent symptom in caregivers of persons with dementia [28,29]. Similar to previous studies [30,31] we have found a higher score of depression in female proxies compared to male proxies. It is beyond the scope of the present manuscript to analyze modulator variables in detail. It is known however, that the well-being and mood of informants may impose a bias on the results obtained with subjective ratings such as ADLs and iADLs [32,33]. It was the primary goal of the present manuscript to analyze substituted QoL judgment and its modulation by caregiver characteristics. Correlational analysis for the single items of the QoL-AD when answered by the proxy for both, himself and herself as well as when answered as a substitute for the patient clearly shows that proxies indeed rated differently when answering for themselves and the patient.

### Proxy-self judgment of the proxie's QoL (proxy(QoL-AD)proxy)

The self-assessed quality of life of the proxy (proxy(QoL-AD)proxy) in the present study was unrelated to severity of dementia. With advancing dementia severity, not only cognitive functions deteriorate but the prevalence and severity of behavioural and psychological symptoms of dementia (BPSD) increase, e.g. agitation. Previous studies reported that these symptoms are significantly associated with caregiver assessment of the patient's QoL but not with patients' self-assessed QoL [34,35]. Univariate analysis of BPSD and the proxies' QoL using a different scale for assessment of BPSD, the Behave-AD, yields a clear-cut association of caregiver QoL (proxy(QoL-AD)proxy) and behavioural symptoms of dementia patients. Likewise, QoL of caregivers (proxy(QoL-AD)proxy) is inversely correlated with decline of basic and instrumental activities of daily living. As expected, there is an association between QoL (proxy(QoL-AD)proxy) in female proxies and the subjective burden imposed by caring for a patient with dementia. However, this association was not found in males. The reasons for this gender-dependency remains elusive on grounds of the current data and needs to be addressed in future studies.

Similar to a previous study [36], depression is higher in female than in male caregivers and the subjective QoL (proxy(QoL-AD)proxy) is worse. Considering similar cognitive impairment and behavioural symptoms in the respective patients, this argues for gender-dependent variables on the proxies' side which cause the differing sense of burden and impairment on quality of life imposed by the stress of caregiving.

### Substituted judgment of the patient's QoL (proxy(QoL-AD substituted)patient)

Patients' self assessment of their own qualiy of life (EQ-5D+C) is impaired even in mild stages of dementia [2,3,37]. Likewise, assessment of the quality of life by the proxy is under discussion [2,12,32,38]. In a previous study, caregiver's judgement of the patient's QoL was associated with the severity of the patient's neuropsychiatric symptoms in general and depressive symptoms in particular [34]. Moreover, dementia severity and the caregiver's depressive mood negatively affect the caregivers' assessments of the patient's QoL [12]. One possible alternative to be considered beyond staging disease severity and disease impact with medical outcome variables, is to instruct the proxy to assume the role of the patient cared for, and to give the appraisal that the patient would have given hadhe or she been competent to do so - substituted judgment. Until now, little is known about substituted judgment on the patients' QoL in patients with dementia.

The present analysis reveals that substituted judgment of the patients' QoL (proxy(QoL-AD substituted)patient) is modulated by proxy-related variables in a gender-dependent fashion. The stepwise regression analysis performed in this study shows that substituted judgment (proxy(QoL-AD substituted)patient) is subject to the self-assessed depressive mood of the patient (patient(GDS)patient), the proxies' assessment of behavioural symptoms of the patient and the subjective QoL of the proxy (proxy(QoL-AD)proxy). In a gender-specific analysis, subjective QoL of the proxy (proxy(QoL-AD)proxy) is the most important variable influencing female caregivers while it is the depression of the patient in male caregivers.

Similarly, gender-dependent aspects were found in a factorial analysis of the QoL-AD. The literature shows two factor analytic analyses for the QoL-AD. One was performed in patients with Alzheimer's disease [24] and the other in healthy elderly individuals [25]. The latter indicated the best fit for a three-factor solution, i.e. physical, social, and psychological well-being [25]. Applying standard factor-analytic procedures and using the Kaiser criterion, the present study also shows the best overall fit for a three-factor solution in proxies of patients with Alzheimer's disease. Overall, there is good agreement for the factor structure of the self-assessment of the proxies' own QoL (proxy(QoL-AD)proxy) and the substituted judgment of the patients' QoL (proxy(QoL-AD substituted)patient). Gender-specific analysis, however, reveals slight differences. Female proxies have an even greater agreement for the factor structure judging their own QoL (proxy(QoL-AD)proxy) and substituted judgment on the patients' QoL(proxy(QoL-AD substituted)patient). Male proxies show some differences: the factor structure for substituted judgment (proxy(QoL-AD substituted)patient) convey factors that may be labelled as general-and health-related, social, and marriage. In contrast, female proxies portray a factor structure which may be better labelled as general- and health-related, social, and functional.

## Conclusion

Substituted judgment of the patient's QoL (proxy(QoL-AD substituted)patient) does not correlate with dementia severity. Substituted judgment is subject to proxy-related variables in a gender-dependent fashion and is therefore not suited to serve as an appropriate surrogate of the patients' quality of life. Even the proxies' self-assessment of their own QoL (proxy(QoL-AD)proxy) is no surrogate for representing deterioration of the patients' well being in patients with dementia.

## Competing interests

The present study was funded by the German Federal Ministry of Health (MNG-LTDEMENZ_04_61). The sponsor did not influence design of the study, analysis of the data, or drafting of the manuscript. CJ, CL and CS report no conflict of interest. FM and MWR have received grants or funding and honoria from companies selling or developing medical products for use in patients with Alzheimers's disease. None of the authors report personal or financial conflicts of interest.

## Authors' contributions

CJ, CL, and CS were involved in acquisition of the data, data analysis, and drafting and revising the manuscript. FM and MWR were involved in designing the study, interpretation of the data, and drafting and revising the manuscript. All authors approved the final version of the manuscript.

## Pre-publication history

The pre-publication history for this paper can be accessed here:

http://www.biomedcentral.com/1471-2377/11/118/prepub
